# A cross-sectional study of retinal vessel changes based on optical coherence tomography angiography in Alzheimer’s disease and mild cognitive impairment

**DOI:** 10.3389/fnagi.2023.1101950

**Published:** 2023-04-11

**Authors:** Xiaoyu Ma, Zengmai Xie, Huan Wang, Zhongping Tian, Yanlong Bi, Yunxia Li, Li Zhang

**Affiliations:** ^1^Department of Ophthalmology, Tongji Hospital, School of Medicine, Tongji University, Shanghai, China; ^2^Department of Neurology, Tongji Hospital, School of Medicine, Tongji University, Shanghai, China; ^3^Clinical Research Center, Tongji Hospital, School of Medicine, Tongji University, Shanghai, China; ^4^Tongji Eye Institute, School of Medicine, Tongji University, Shanghai, China

**Keywords:** optical coherence tomography angiography, Alzheimer’s disease, mild cognitive impairment, vessel density, perfusion density, cognitive function

## Abstract

**Background:**

The involvement of retina and its vasculature has been recently described in Alzheimer’s disease (AD). Optical coherence tomography angiography (OCTA) is noninvasively used to assess the retinal blood flow.

**Objective:**

This study was to compare vessel density (VD) and blood perfusion density (PD) of the macular in AD patients, mild cognitive impairment (MCI) patients and healthy controls by OCTA, which may provide new ideas for diagnosis of AD or MCI.

**Methods:**

AD patients, MCI patients and healthy controls underwent a comprehensive ophthalmic and neurological evaluations, including cognitive function assessments as well as visual acuity, intraocular pressure (IOP), slit lamp examinations, and OCTA. General demographic data, cognitive function, retinal VD and PD were compared among three groups. The correlations among retinal VD, PD and cognitive function, amyloid-beta (Aβ) protein and phosphorylated Tau (p-Tau) protein were further evaluated. The correlations between retinal superficial capillary plexus and cognitive function, Aβ protein and p-Tau protein were also explored.

**Results:**

A total of 139 participants were recruited into this study, including 43 AD patients, 62 MCI patients, and 34 healthy controls. After adjusting for sex, age, history of smoking, history of alcohol intake, hypertension, hyperlipidemia, best corrected visual acuity, and IOP, VD and PD in the nasal and inferior regions of the inner ring, superior and inferior regions of outer ring in the AD group were significantly lower than in the control group (*p* < 0.05). PD in nasal region of outer ring also significantly decreased in the AD group. VD and PD in superior and inferior regions of inner ring, superior and temporal regions of outer ring in the MCI group were markedly lower than in the control group (*p* < 0.05). After adjusting for sex and age, VD and PD were correlated with Montreal Cognitive Assessment Basic score, Mini-mental State Examination score, visuospatial function and executive function (p < 0.05), while Aβ protein and p-Tau protein had no relationship with VD and PD.

**Conclusion:**

Our findings suggest that superficial retinal VD and PD in macula may be potential non-invasive biomarkers for AD and MCI, and these vascular parameters correlate with cognitive function.

## Introduction

Alzheimer’s disease (AD) is a common progressive degenerative disease of the central nervous system, and has been the most common cause of dementia, accounting for 50–75% (Lane et al., 2018). It has a high prevalence in the elderly and early old age, and is mainly characterized by progressive cognitive dysfunction and behavioral impairment (Lane et al., 2018). Mild cognitive impairment (MCI) is the transitional stage between normal aging and dementia (Gauthier et al., 2006). It is characterized by cognitive decline but the ability to live a normal life is not affected (Kim et al., 2017). Of note, people with MCI have a high risk of developing dementia. It is estimated that 32% of MCI patients will develop AD within the next 5 years (Ward et al., 2013). AD patients bring great economic and social burdens to the family and even the whole of society. It is estimated that the annual total cost of nursing AD patients is more than US $507.49 billion in 2030 in China (Jia et al., 2018). Prevention and treatment of AD are still a worldwide problem. These may be ascribed to the difficult early diagnosis of AD. Because its onset is insidious, with pathological changes in the brain occurring 20 years or more before clinical symptoms (Villemagne et al., 2013; Gordon et al., 2018). The identification of these pathological changes in the brain requires expensive positron emission tomography/computed tomography (PET-CT) and invasive cerebrospinal fluid (CSF) tests, which are not widely available in clinical practice (Jack et al., 2018). Therefore, it is imperative to develop economical and noninvasive tests for the early recognition of AD and MCI.

The retina and the brain share some features in embryology, anatomy and physiology (Lee et al., 2020). First, the retina develops from the neuroectoderm, having the same embryonic origin as the brain, and is a sensory extension of the brain (Hart et al., 2016). Secondly, the retina is an extension of the diencephalon and has a blood-retinal barrier similar to the blood–brain barrier (Baker et al., 2008). Retinal small blood vessels and small cerebral blood vessels also have similar physiological properties (Patton et al., 2005). The microcirculation systems of both are hyperoxic extraction systems, and their blood flow depends on regional neuronal activity (Patton et al., 2005). The automatic regulation mechanism makes the perfusion pressure of the vessels maintain relatively constant blood flow even if it changes (Yan et al., 2021). Moreover, autopsy has indicated the amyloid-beta (Aβ) protein deposits in the retinal vessels of AD patients (Shi et al., 2020). This suggests that retinal vascular disease can objectively reflect the vascular disease in the brain, and is a window to study cerebral vasculopathy (Newman, 2013).

Studies have revealed that changes in brain perfusion exist long before the clinical symptoms of AD, and may even predate Aβ protein accumulation or brain shrinkage (Hays et al., 2016). However, the changes of blood flow in the brain cannot be directly observed. Based on the similarity between the retina and the brain, it is possible to detect the blood flow in the retina to reflect the changes of blood flow in the brain. Optical coherence tomography angiography (OCTA) is a non-invasive, rapid and high-resolution fundus angiography technique, which can observe the structure and morphology of blood vessels at different levels of the retina in layers, and quantify the blood flow index and diseased blood flow area within a certain range (Boeckaert et al., 2012). OCTA can be used to collect information on blood vessel density (VD) and blood vessel morphology of the retina in macular area. Studies have indicated that, compared with normal controls, the blood VD in the superficial and deep retina of macular area in AD and MCI patients significantly reduced, and the foveal avascular zone (FAZ) area was significantly enlarged, which is a sign of macular ischemia (Bulut et al., 2018; Jiang et al., 2018; Lahme et al., 2018; Zabel et al., 2019). In addition, the fractal dimension (FD) of the superficial vascular network also significantly reduced in AD patients (Chua et al., 2020), while FD reflected the complexity of retinal vascular branches and the density of the entire retinal vascular system. However, the changes in FD in MCI patients remain controversial. One study shows that FD in the superficial vascular network significantly reduces in MCI patients as compared to normal controls (Chua et al., 2020). But another case–control study shows a significant increase in the retinal FD in patients with MCI due to AD (Biscetti et al., 2021). There are also some changes in the choroid in AD and MCI patients. Compared with normal controls, choroid thickness was significantly thinner in AD patients (Trebbastoni et al., 2017; Salobrar-Garcia et al., 2020). However, the choroid thickness of MCI patients tends to become thinner although there is no statistical significance (López-de-Eguileta et al., 2020).

Therefore, this study was to compare VD and blood perfusion density (PD) of macular retinal superficial capillary plexus (SCP) in AD patients, MCI patients and healthy controls by OCTA. The relationships among the retinal microvascular network and cognitive function, Aβ protein and phosphorylated Tau (p-Tau) protein were also investigated.

## Materials and methods

### Participants

This study design was approved by the Clinical Research Ethics Committee of Tongji Hospital, Shanghai [(Tong) Audit No. (K-2017-003-XZ-190130)], and conducted according to the Declaration of Helsinki. All participants signed informed consent forms before the study. Inclusion criteria: (1) Patients were 50–90 years; (2) Patients were diagnosed with AD or MCI; (3) Scanning signal intensity index on OCTA was >4; (4) Healthy controls (HC) had no history of dementia and had normal cognitive function. Exclusion criteria: (1) History of diabetes mellitus, uncontrolled hypertension, heart disease, or other serious chronic medical conditions; (2) Refractive error > ±6 spherical equivalent; (3) Intraocular surgery within 6 months; (4) A history of glaucoma or intraocular pressure (IOP) >21 mmHg; (5) Macular disease or retinopathy, such as age-related macular degeneration, macular anterior membrane, retinal vascular obstruction, etc.; (6) Other neurological diseases or severe psychiatric illnesses. (7) Apparent media opacification.

### Diagnosis

The study participants were all from the Department of Neurology or Ophthalmology of Tongji Hospital in Shanghai. Data were collected from July 2020, to August 2022. The diagnoses of AD and MCI was based on 2011 guidelines of the National Institute of Aging-Alzheimer’s Association workgroups (NIA/AA) (McKhann et al., 2011) and the quantitative criteria proposed by Jak/Bondi in 2014 (Bondi et al., 2014).

Alzheimer’s disease: (1) Insidious onset and slow progression of symptoms; (2) A clear history of cognitive deterioration; (3) Impaired ability to function in daily life; (4) Cognitive impairment was classified into the following categories when the medical history and neuropsychological assessment were reviewed: (a) Amnestic presentation; (b) Nonamnestic presentations: language disorders, visuospatial disorders, and executive dysfunction; (5) Exclusion of other causes of dementia, such as metabolic disorder and encephalopathy.

Mild cognitive impairment: (1) Cognitive concern reflecting a change in cognition reported by the patient or relatives or clinicians; (2) Mini-Mental State Examination (MMSE) scores: illiterate ≤17, elementary school ≤20, middle school and above ≤24; or Montreal Cognitive Assessment Basic (MoCA-B) scores: elementary school and below ≤19, secondary school ≤22, college ≤24; (3) Clinical Dementia Rating Scale (CDR) = 0.5, not enough to diagnose dementia; (4) Meeting any one of the following three criteria: (a) impairment of 2 metrics in the same cognitive domain [score are 1 standard deviations (SD) below the mean for their age and education matched peers]; (b) impairment of 1 test score in 2 or more of the four cognitive domains (score are 1 SD below the mean for their age and education matched peers); (c) instrumental activities of daily living (IADL) score: more than one item score of 1 or more.

### Neurological and ophthalmic examinations

All participants underwent neurological tests. A full set of cognitive scales were used to assess their cognitive status, including: MoCA-B, MMSE, IADL, Hamilton anxiety scale (HAMA), Hamilton depression scale (HAMD), Hopkins Verbal Learning Test (HVLT), Wechsler memory scale (WMS), Boston naming test (BNT), Verbal fluency test (VFT), Shape trails test (STT), and Rey-Osterrieth complex figure test (ROCFT). In addition, medical history, and results from laboratory and neuroimaging examinations were collected to aid the diagnosis. Furthermore, with the consent of some participants, CSF was collected and tested for Aβ and p-Tau proteins. CSF samples were collected from 23 patients, including 16 AD patients and 7 MCI patients.

A complete ophthalmic examination was administered, including the measurement of best-corrected visual acuity (BCVA), IOP, slit lamp examination and conventional OCTA of the macula. An international standard logarithmic visual acuity chart was used to measure the BCVA. IOP was measured three times with a hand-held tonometer, and the average value was taken. OCTA images and slit lamp examination were used to rule out other eye diseases.

### Procedures for OCTA

**Figure 1:**
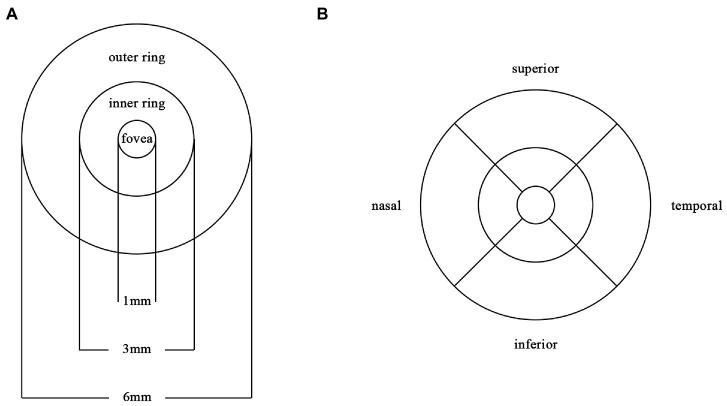
Partition diagram Partition diagram of the macula. **(A)** The macula is divided into 1×1mm fovea, 3×3mm inner ring and 6×6mm outer ring. **(B)** The inner ring and outer ring are divided into superior, inferior, nasal and temporal subregions.

The ZEISS Angioplex™ OCTA (Carl Zeiss Meditec, Dublin, CA) was used to scan the macula of all the participants. It has a scan rate of 68,000 A-scans per second, a central wavelength of 840 nm, and motion tracking to reduce motion artifacts. 6 × 6 mm images centered on the fovea were acquired. This study focuses on retinal SCP, which was defined as the area from the internal limiting membrane (ILM) to the inner plexiform layer (IPL). We subdivided the macula into 1 × 1 mm fovea subregion, 3 × 3 mm inner ring and 6 × 6 mm outer ring. Meanwhile, the inner ring and outer ring were divided into superior, inferior, nasal and temporal subregions (Figure 1). VD was defined as the ratio of total length of blood vessels in the region to the area of the region, whereas PD was defined as the ratio of covered area of blood vessels in the region to the area of the region. The built-in software automatically calculates the area of the macular foveal avascular zone (FAZ), VD and PD.

All the examinations are performed by the same skilled clinicians. The clinician input the patient’s name, gender and date of birth, and informed the patient of the precautions for examination to relieve the patient’s nervousness and clear images were captured. Participants were seated with their mandible placed on the mandibular support and their forehead pressed against the front support. The position was adjusted so that the patient’s lateral canthus was at the same height as the horizontal line. Good fixation is required during the scanning. After each scan, the operator determines whether rescanning is needed depending on the image quality. The high-quality images were captured and saved in the computer.

### Statistical analysis

The enumeration data are represented by the number of cases, and the Chi-square test was used for comparisons between groups. The measurement data are expressed as mean ± standard deviation, and Shapiro–Wilk test was used to test the normality of these data. If the data were normally distributed, one-way analysis of variance (ANOVA) was used for comparisons among groups. If the data were not normally distributed, Kruskal–Wallis *H* method was used for comparisons between groups. Multiple logistic regression models for each of the OCTA parameters with adjustments for confounding factors were used to compare AD, MCI and HC subjects. Partial correlation analysis was used to evaluate the correlations among OCTA parameters and neuropsychological assessment scores, Aβ and p-Tau protein. A value of *p* < 0.05 was considered statistically significant. Statistical analysis was performed using Statistical Package for Social Sciences (version 20.0, SPSS Inc., Chicago, IL, United States).

## Results

### Patient characteristics

A total of 139 subjects were included in this study, including 43 AD patients, 62 MCI patients and 34 HC. The eye with high-quality image was selected from each participant. Of AD patients, there were 21 (48.8%) males, and 58.1% were older than 70 years. Of MCI patients, there were 25 (40.3%) males, and 53.2% were older than 70 years. Of healthy controls, there were 18 (52.9%) males, and 50% were older than 70 years. The demographic characteristics of AD patients, MCI patients, and HC are shown in Table 1. There were no significant differences in the age, sex, history of smoking, history of alcohol intake, hypertension, hyperlipidemia, years of education, IOP and FAZ among AD, MCI and HC groups (*p* > 0.05). The BCVA was 0.63 ± 0.26 in the AD group, 0.74 ± 0.22 in the MCI group, and 0.76 ± 0.23 in the HC group (*p* = 0.037).

**Table 1:** Patient’s demographic characteristics.

	AD (*n* = 43)	MCI (*n* = 62)	HC (*n* = 34)	*P*
Sex				0.449
Female	22(51.2%)	37(59.7%)	16(47.1%)	
Male	21(48.8%)	25(40.3%)	18(52.9%)	
Age				0.767
50–70 years	18(41.9%)	29(46.8%)	17(50%)	
>70 years	25(58.1%)	33(53.2%)	17(50%)	
History of smoking (Yes)	11(25.6%)	13(21%)	11(32.3%)	0.469
History of alcohol intake (Yes)	3(6.9%)	2(3.2%)	1(3.0%)	0.608
Hypertension (Yes)	15(34.9%)	24(37.5%)	13(38.2%)	0.918
Hyperlipidemia (Yes)	12(27.9%)	11(17.7%)	13(38.2%)	0.085
Years of education	10.3 ± 5.32	11.05 ± 4.15	10.68 ± 3.86	0.76
BCVA	0.63 ± 0.26	0.74 ± 0.22	0.76 ± 0.23	0.037*
IOP	14.12 ± 1.80	14.4 ± 1.84	14.53 ± 1.75	0.349
FAZ	0.34 ± 0.14	0.35 ± 0.14	0.34 ± 0.15	0.978

Data are shown as mean ± standard deviation or number. AD, Alzheimer’s disease; MCI, mild cognitive impairment; HC, healthy controls; F, female; M, male; BCVA, best-corrected visual acuity; IOP, intraocular pressure; FAZ, foveal avascular zone. *Significant at *p* < 0.05. The bold values indicate that the number is < 0.05, indicating a statistical difference.

The scores of neuropsychological assessments in each group are shown in Table 2. There was no significant difference in the HAMA score among three groups (*p* = 0.089). There were significant differences in the MMSE score (*p* < 0.001), MoCA-B score (*p* < 0.001), HAMD score (*p* = 0.009), HVLT score (*p* < 0.001), WMS score (*p* < 0.001), BNT score (p < 0.001), VFT score (*p* < 0.001), STT score (*p* < 0.001) and ROCFT score (*p* < 0.001) among AD, MCI and HC groups.

**Table 2:** Assessment of cognitive function in each group.

	AD (*n* = 43)	MCI (*n* = 62)	HC (*n* = 34)	*P*
MMSE	16.56 ± 6.68	25.19 ± 2.48	27.15 ± 2.23	<0.001*
MoCA-B	10.67 ± 6.09	19.23 ± 4.68	22.85 ± 3.78	<0.001*
HAMD	9.28 ± 6.29	7.90 ± 4.95	5.41 ± 4.31	0.009*
HAMA	9.67 ± 6.42	9.02 ± 5.82	6.62 ± 4.79	0.089*
Memory function				
HVLT- immediate	2.49 ± 1.76	4.06 ± 1.58	4.47 ± 1.58	<0.001*
HVLT-5 min delay	0.84 ± 2.06	4.45 ± 3.38	6.65 ± 2.87	<0.001*
HVLT-20 min delay	0.74 ± 2.18	4.03 ± 3.35	6.74 ± 2.80	0.001*
WMS	3.98 ± 2.69	7.03 ± 2.93	8.71 ± 2.74	<0.001*
Language function				
BNT	16.09 ± 6.71	21.56 ± 3.78	23.76 ± 3.39	<0.001*
VFT	7.21 ± 3.28	11.76 ± 3.29	14.32 ± 3.19	<0.001*
Executive function				
STT-1	95.67 ± 47.55	67.84 ± 25.94	56.03 ± 17.30	<0.001*
STT-2	182.08 ± 48.02	155.53 ± 47.48	135.29 ± 39.97	0.001*
Visuospatial function				
ROCFT-copy	22.35 ± 13.91	32.92 ± 4.73	34.88 ± 1.49	<0.001*
ROCFT-recall	4 ± 7.65	11.6 ± 7.96	18.06 ± 6.79	<0.001*

Data are shown as mean ± standard deviation or number. AD, Alzheimer’s disease; MCI, mild cognitive impairment; HC, healthy controls; MMSE, Mini-mental State Examination; MoCA-B, Montreal Cognitive Assessment Basic; HAMD, Hamilton depression scale; HAMA, Hamilton anxiety scale; HVLT, Hopkins Verbal Learning Test; WMS, Wechsler memory scale; BNT, Boston naming test; VFT, Verbal fluency test; STT, Shape trails test; ROCFT, Rey-Osterrieth complex figure test. *Significant at *p* < 0.05. The bold values indicate that the number is < 0.05, indicating a statistical difference.

### Vessel density

**Figure 2:**
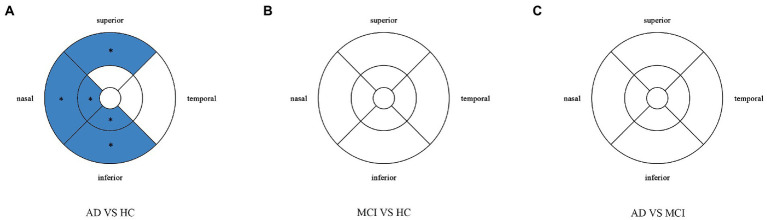
Comparison of vessel density in macular among the AD, MCI, and HC groups. Blue regions indicate that the vessel density of the former decreases significantly in these areas compared to the latter. **(A)** In the AD group, the vessel density significantly reduces in the nasal and inferior regions of inner ring, and in the nasal, superior and inferior regions of outer ring as compared to HC group. **(B,C)** There is no significant difference between MCI group and HC group, and between AD group and MCI group. *Significant at *p* < 0.05. AD, Alzheimer’s disease; MCI, mental cognitive impairment; HC, healthy controls.

**Figure 3:**
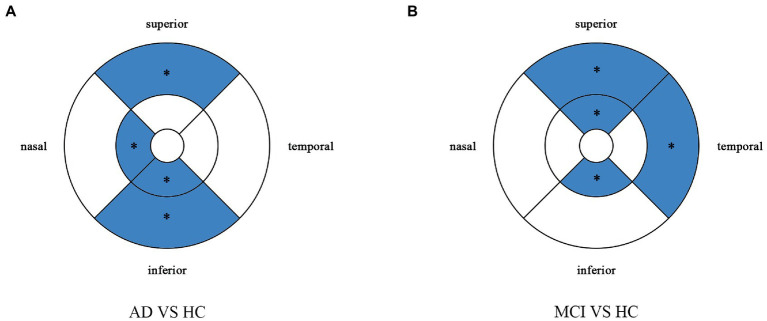
Multiple logistic regression was used to assess the association between retinal vessel density and clinical diagnosis, adjusted for confounders of sex, age, history of smoking, history of alcohol intake, hypertension, hyperlipidemia, BCVA, and IOP. Blue regions indicate decreases significantly compared with the HC group. **(A)** Compared with HC group, vessel density decreases significantly in the nasal and inferior regions of the inner ring and in the superior and inferior regions of the outer ring in the AD group. **(B)** Compared with HC group, vessel density decreases significantly in the superior and inferior regions of the inner ring and in the temporal and superior regions of the outer ring in the MCI group. *Significant at p < 0.05. AD, Alzheimer’s disease; MCI, mental cognitive impairment; HC, healthy controls.

**Figure 4:**
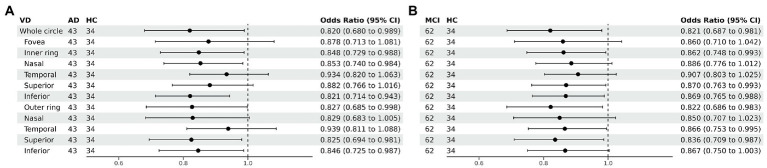
Forest plot of vessel density comparison among three groups. Multiple logistic regression was used to access the association between retinal vessel density and clinical diagnosis, adjusted for confounders of sex, age, history of smoking, history of alcohol intake, hypertension, hyperlipidemia, BCVA, and IOP. **(A)** Compared with HC group, vessel density decreased significantly in the nasal and inferior regions of the inner ring and in the superior and inferior regions of the outer ring in the AD group. **(B)** Compared with HC group, vessel density decreased significantly in the superior and inferior regions of the inner ring and in the temporal and superior regions of the outer ring in the MCI group. VD, vessel density; AD, Alzheimer’s disease; MCI, mental cognitive impairment; HC, healthy controls; CI, confidence interval.

Compared with HC group (14.13 ± 3.00), the VD of the whole circle region (12.26 ± 2.87) significantly decreased in the AD group (*p* < 0.05). The whole circle was divided into three regions: fovea subregion, inner ring and outer ring. The results showed that, compared with HC group, the VD in the inner ring (AD:10.74 ± 3.42, HC:13.02 ± 3.70, *p* < 0.05) and outer ring (AD:13.07 ± 2.92, HC:14.83 ± 2.91, *p* < 0.05) regions significantly decreased in the AD group. Then, the inner ring and the outer ring were divided into four regions, respectively (Figure 1). In the AD group, the VD also significantly reduced in the nasal (AD:10.96 ± 3.79, HC:13.43 ± 4.12, *p* = 0.008) and inferior (AD:9.66 ± 4.03, HC:12.95 ± 3.80, *p* = 0.001) regions of inner ring, nasal (AD:15.72 ± 2.87, HC:17.39 ± 2.95, *p* = 0.011), superior (AD:12.99 ± 3.12, HC: 15.00 ± 3.03, *p* = 0.009) and inferior (AD: 12.2 ± 3.78, HC: 14.48 ± 3.39, *p* = 0.017) region of outer ring as compared to HC group (Table 3 and Figure 2). There were no significant differences in other areas. There was no significant difference between MCI group and HC group, and between AD group and MCI group (*p* > 0.05) (Table 3 and Figure 2). After adjusting for confounding factors such as sex, age, history of smoking, history of alcohol intake, hypertension, hyperlipidemia, BCVA, and IOP, multiple logistic regression analysis showed that the significant differences remained in the AD group except for the nasal (OR = 0.829, 95%CI: 0.683, 1.005) region of outer ring (Figures 3, 4). Compared with HC group, VD decreased significantly in the nasal (OR = 0.853, 95%CI: 0.740, 0.984) and inferior (OR = 0.821, 95%CI: 0.714, 0.943) regions of the inner ring and in the superior (OR = 0.825, 95%CI: 0.694, 0.981) and inferior (OR = 0.846, 95%CI: 0.725, 0.987) regions of the outer ring in the AD group (Figures 3, 4). Compared with HC group, VD also decreased significantly in the superior (OR = 0.870, 95%CI:0.763, 0.993) and inferior (OR = 0.869, 95%CI: 0.765, 0.988) regions of the inner ring and in the temporal (OR = 0.866, 95%CI: 0.753, 0.995) and superior (OR = 0.836, 95%CI: 0.709, 0.987) regions of the outer ring in the MCI group (Figures 3, 4).

**Table 3:** Comparison of vessel density in macular among the three groups.

VD	AD (*n* = 43)	MCI (*n* = 62)	HC (*n* = 34)	*P*	AD vs. HC	MCI vs. HC	AD vs. MCI
					*P*	*P*	*P*
Whole circle	12.26 ± 2.87	12.68 ± 3.36	14.13 ± 3.00	0.011*	0.009*	0.085	0.872
Fovea	3.2 ± 2.24	3.43 ± 2.22	4.32 ± 2.91	0.239	>0.05	>0.05	>0.05
Inner ring	10.74 ± 3.42	11.35 ± 3.73	13.02 ± 3.70	0.013*	0.012*	0.078	1.000
Nasal	10.96 ± 3.79	11.77 ± 3.68	13.43 ± 4.12	0.009*	0.008*	0.068	0.887
Temporal	11.1 ± 3.66	11.28 ± 4.34	12.68 ± 4.04	0.172	>0.05	>0.05	>0.05
Superior	11.18 ± 3.62	11.33 ± 3.94	13.02 ± 3.92	0.07	>0.05	>0.05	>0.05
Inferior	9.66 ± 4.03	11.03 ± 4.07	12.95 ± 3.80	0.002*	0.001*	0.065	0.321
Outer ring	13.07 ± 2.92	13.42 ± 3.37	14.83 ± 2.91	0.018*	0.017*	0.091	1.000
Nasal	15.72 ± 2.87	16.15 ± 3.33	17.39 ± 2.95	0.013*	0.011*	0.086	0.958
Temporal	11.31 ± 3.26	10.97 ± 4.06	12.46 ± 3.64	0.184	>0.05	>0.05	>0.05
Superior	12.99 ± 3.12	13.54 ± 3.67	15.00 ± 3.03	0.011*	0.009*	0.135	0.585
Inferior	12.2 ± 3.78	12.94 ± 3.77	14.48 ± 3.39	0.019*	0.017*	0.114	1.000

Data are shown as mean ± standard deviation or number. VD, vessel density; AD, Alzheimer’s disease; MCI, mild cognitive impairment; HC, healthy controls. *Significant at *p* < 0.05. The bold values indicate that the number is < 0.05, indicating a statistical difference.

### Perfusion density

**Figure 5:**
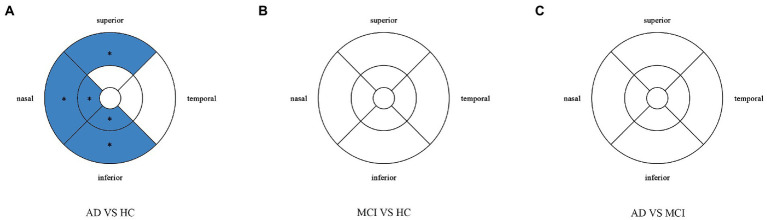
Comparison of perfusion density in macular among the AD, MCI, and HC groups. Blue regions indicate that the perfusion density of the former decreases significantly in these areas compared to the latter. **(A)** In the AD group, the perfusion density significantly reduces in the nasal and inferior regions of inner ring, and in the nasal, superior snd inferior regions of outer ring as compared to HC group. **(B,C)** There is no significant difference between MCI group and HC group, and between AD group and MCI group. *Significant at *p* < 0.05. AD, Alzheimer’s disease; MCI, mental cognitive impairment; HC, healthy controls.

**Figure 6:**
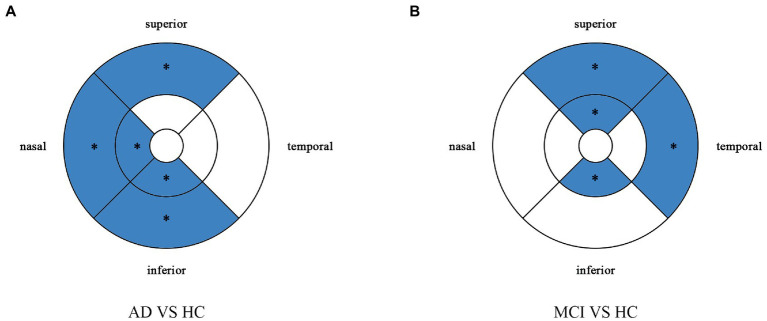
Multiple logistic regression was used to assess the association between retinal perfusion density and clinical diagnosis, adjusted for confounders of sex, age, history of alcohol intake, hypertension, hyperlipidemia, BCVA, and IOP. Blue regions indicate decreases significantly compared with the HC group. **(A)** Compared with HC group, perfusion density decreases significantly in the nasal and inferior regions of the inner ring and in the nasal, superior and inferior regions of the outer ring in the AD group. **(B)** Compared with HC group, perfusion density decreases significantly in the superior and inferior regions of the inner ring and in the temporal and superior regions of the outer ring in the MCI group. *Significant at *p* < 0.05. AD, Alzheimer’s disease; MCI, mental cognitive impairment; HC, healthy controls.

**Figure 7:**
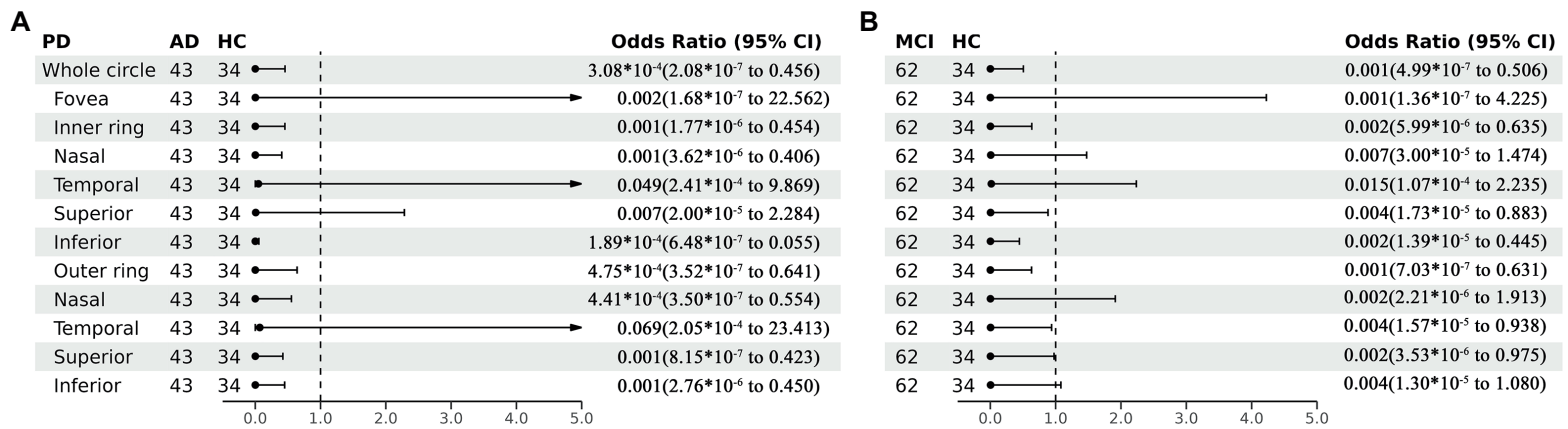
Forest plot of perfusion density comparison among three groups. Multiple logistic regression was used to assess the association between retinal perfusion density and clinical diagnosis, adjusted for confounders of sex, age, history of smoking, history of alcohol intake, hypertension, hyperlipidemia, BCVA, and IOP. **(A)** Compared with HC group, perfusion density decreased significantly in the nasal and inferior regions of the inner ring and in the nasal, superior and inferior regions of the outer ring in the AD group. **(B)** Compared with HC group, perfusion density decreased significantly in the superior and inferior regions of the inner ring and in the temporal and superior regions of the outer ring in the MCI group. PD, perfusion density; AD, Alzheimer’s disease; MCI, mental cognitive impairment; HC, healthy controls; CI, confidence interval.

Perfusion density showed the same results to VD. Compared with HC group (0.338 ± 0.077), the PD (0.29 ± 0.07) in the whole circle region significantly decreased in the AD group (*p* < 0.05). The whole circle was divided into three regions. Compared with HC group, the PD in the inner ring (AD: 0.24 ± 0.08, HC: 0.302 ± 0.093, *p* < 0.05) and outer ring (AD: 0.31 ± 0.07, HC: 0.358 ± 0.076, *p* < 0.05) regions significantly decreased in the AD group. Then, the inner and outer rings were further explored. In the AD group, the PD also significantly reduced in nasal (AD: 0.25 ± 0.09, HC: 0.309 ± 0.102, *p* = 0.007) and inferior (AD: 0.22 ± 0.1, HC: 0.305 ± 0.097, *p* = 0.001) regions of inner ring, nasal (AD: 0.37 ± 0.08, HC: 0.421 ± 0.078, *p* = 0.006), superior (AD: 0.31 ± 0.08, HC: 0.364 ± 0.080, p = 0.007) and inferior (AD: 0.29 ± 0.1, HC: 0.351 ± 0.087, *p* = 0.017) regions of outer ring as compared to the HC group (Table 4 and Figure 5). There were no significant differences in other areas. There was no significant difference between MCI group and HC group, and between AD group and MCI group (*p* > 0.05) (Table 4 and Figure 5). After adjusting for confounding factors such as sex, age, history of smoking, history of alcohol intake, hypertension, hyperlipidemia, BCVA, and IOP, multiple logistic regression analysis showed that significant differences remained in the AD group. Compared with HC group, VD decreased significantly in the nasal (OR = 0.001, 95%CI: 3.62*10^−6^, 0.406) and inferior (OR = 1.89*10^−4^, 95%CI: 6.48*10^−7^, 0.055) regions of the inner ring and in the nasal (OR = 4.41*10^−4^, 95%CI: 3.50*10^−7^, 0.554), superior (OR = 0.001, 95%CI: 8.15*10^−7^, 0.423) and inferior (OR = 0.001, 95%CI: 2.76*10^−6^, 0.450) regions of the outer ring in the AD group (Figures 6, 7). Compared with HC group, VD also decreased significantly in the superior (OR = 0.004, 95%CI: 1.73*10^−5^, 0.883) and inferior (OR = 0.002, 95%CI: 1.39*10^−5^, 0.445) regions of the inner ring and in the temporal (OR = 0.004, 95%CI: 1.57*10^−5^, 0.938) and superior (OR = 0.002, 95%CI: 3.53*10^−6^, 0.975) regions of the outer ring in the MCI group (Figures 6, 7).

**Table 4:** Comparison of perfusion density in macular among the three groups.

PD	AD (*n* = 43)	MCI (*n* = 62)	HC (*n* = 34)	*P*	AD vs. HC	MCI vs. HC	AD vs. MCI
					*P*	*P*	*P*
Whole circle	0.29 ± 0.07	0.3 ± 0.08	0.338 ± 0.077	0.009*	0.007*	0.092	0.676
Fovea	0.07 ± 0.05	0.07 ± 0.05	0.093 ± 0.066	0.218	>0.05	>0.05	>0.05
Inner ring	0.24 ± 0.08	0.26 ± 0.09	0.302 ± 0.093	0.012*	0.01*	0.078	0.970
Nasal	0.25 ± 0.09	0.27 ± 0.09	0.309 ± 0.102	0.009*	0.007*	0.078	0.798
Temporal	0.25 ± 0.09	0.26 ± 0.1	0.293 ± 0.101	0.144	>0.05	>0.05	>0.05
Superior	0.26 ± 0.09	0.26 ± 0.1	0.302 ± 0.096	0.076	>0.05	>0.05	>0.05
Inferior	0.22 ± 0.1	0.25 ± 0.1	0.305 ± 0.097	0.001*	0.001*	0.062	0.266
Outer ring	0.31 ± 0.07	0.32 ± 0.09	0.358 ± 0.076	0.017*	0.014*	0.116	0.897
Nasal	0.37 ± 0.08	0.39 ± 0.09	0.421 ± 0.078	0.008*	0.006*	0.091	0.647
Temporal	0.27 ± 0.08	0.26 ± 0.1	0.297 ± 0.093	0.194	>0.05	>0.05	>0.05
Superior	0.31 ± 0.08	0.33 ± 0.1	0.364 ± 0.080	0.01*	0.007*	0.194	0.374
Inferior	0.29 ± 0.1	0.31 ± 0.1	0.351 ± 0.087	0.021*	0.017*	0.163	0.776

Data are shown as mean ± standard deviation or number. PD, perfusion density; AD, Alzheimer’s disease; MCI, mild cognitive impairment; HC, healthy controls. *Significant at *p* < 0.05. The bold values indicate that the number is < 0.05, indicating a statistical difference.

### Correlation analysis

The correlations between OCTA parameters and cognitive function were further evaluated using partial correlation analysis. After adjusting for sex and age, VD and PD of the inner ring region were correlated with MoCA-B score, MMSE score, STT-A score and ROCFT score. VD of the outer ring region was correlated with MoCA-B score and STT-A score. PD of the outer ring region was correlated with MoCA-B score, STT-A score and ROCFT-recall. VD and PD of the fovea region were correlated with MoCA-B score and ROCFT-recall (Table 5).

**Table 5:** Correlation between retinal blood flow and cognitive function.

		VD				PD			
		Whole circle	Fovea	Inner ring	Outer ring	Whole circle	Fovea	Inner ring	Outer ring
MoCA-B	*r*	**0.200**	**0.224**	**0.234**	**0.176**	**0.207**	**0.223**	**0.240**	**0.188**
	*p*	0.019*	0.009*	0.006*	0.039*	0.015*	0.009*	0.005*	0.028*
MMSE	*r*	0.146	0.132	**0.185**	0.122	0.152	0.131	**0.189**	0.132
	*p*	0.088	0.125	0.03*	0.154	0.077	0.127	0.027*	0.123
Memory function									
HVLT-immediate	*r*	0.029	0.009	0.068	0.012	0.029	0.006	0.066	0.015
	*p*	0.739	0.921	0.429	0.892	0.736	0.946	0.445	0.861
HVLT-5 min delay	*r*	0.092	0.112	0.139	0.069	0.099	0.115	0.143	0.08
	*p*	0.285	0.192	0.104	0.426	0.251	0.182	0.096	0.355
HVLT-20 min delay	*r*	0.108	0.127	0.163	0.082	0.115	0.131	0.168	0.092
	*p*	0.208	0.139	0.057	0.339	0.181	0.128	0.05	0.284
WMS	*r*	0.145	0.152	0.159	0.131	0.151	0.152	0.165	0.140
	*p*	0.091	0.077	0.063	0.126	0.077	0.076	0.054	0.102
Language function									
BNT	*r*	0.120	0.099	0.097	0.121	0.128	0.104	0.103	0.131
	*p*	0.164	0.248	0.258	0.159	0.137	0.225	0.229	0.128
VFT	*r*	0.043	0.088	0.077	0.026	0.049	0.09	0.082	0.035
	*p*	0.615	0.307	0.374	0.762	0.568	0.297	0.338	0.688
Executive function									
STT-A	*r*	**−0.226**	−0.158	**−0.208**	**−0.222**	**−0.225**	−0.166	**−0.206**	**−0.223**
	*p*	0.011*	0.076	0.019*	0.013*	0.011*	0.064	0.021*	0.012*
STT-B	*r*	−0.045	−0.149	−0.063	−0.034	0.046	−0.155	0.058	0.039
	*p*	0.633	0.115	0.508	0.723	0.625	0.099	0.538	0.684
Visuospatial function									
ROCFT-copy	*r*	0.163	0.111	**0.168**	0.153	0.168	0.112	**0.173**	0.160
	*p*	0.058	0.196	0.049*	0.074	0.050	0.194	0.043*	0.061
ROCFT-recall	*r*	**0.180**	**0.180**	**0.202**	0.163	**0.183**	**0.180**	**0.203**	**0.169**
	*p*	0.036*	0.036*	0.018*	0.058	0.033*	0.035*	0.017*	0.049*

Partial correlation analysis was used to evaluate the correlations between retinal blood flow and cognitive function, adjusted for confounders of sex, age. VD, vessel density; PD, perfusion density; MMSE, Mini-mental State Examination; MoCA-B, Montreal Cognitive Assessment Basic; HVLT, Hopkins Verbal Learning Test; WMS, Wechsler memory scale; BNT, Boston naming test; VFT, Verbal fluency test; STT, Shape trails test; ROCFT, Rey-Osterrieth complex figure test. *Significant at *p* < 0.05. The bold values indicate that the number is < 0.05, indicating a statistical difference.

The CSF was collected for the detection of Aβ and p-Tau protein. The correlation between OCTA parameters and Aβ or p-Tau protein was further evaluated. After adjusting for sex and age, the results showed no correlation between them (Table 6).

**Table 6:** Correlation between retinal blood flow and Aβ, p-Tau protein.

	Aβ protein (*n* = 23)		p-Tau protein (*n* = 23)	
	*r*	*P*	*r*	*P*
VD				
Fovea	−0.074	0.743	−0.215	0.336
Inner ring	0.009	0.968	−0.305	0.168
Outer ring	0.078	0.730	−0.067	0.767
Whole circle	0.065	0.772	−0.147	0.514
PD				
Fovea	−0.053	0.815	−0.233	0.297
Inner ring	0.012	0.957	−0.318	0.150
Outer ring	0.077	0.734	−0.07	0.759
Whole circle	0.063	0.781	−0.146	0.517

Partial correlation analysis was used to evaluate the correlations between retinal blood flow and Aβ, p-Tau protein, adjusted for confounders of sex, age. Aβ, amyloid-beta protein; p-Tau, phosphorylated Tau protein; VD, vessel density; PD, perfusion density.

## Discussion

In this cross-sectional study, changes in blood vessels and blood flow of the SCP were investigated in patients with AD and MCI. VD and PD of the SCP significantly reduced in patients with AD and MCI compared with healthy controls. This suggests that the retinal microvascular system is damaged in patients with AD and MCI. And these blood flow indicators correlated with cognitive function. However, no correlation was revealed among these blood flow indicators and Aβ, p-Tau protein.

The mechanism underlying the decreased retinal blood flow in AD patients remains unclear. It has been proposed that Aβ protein may be deposited in the retina, causing damage to blood vessels. Autopsy results and AD animal experiments have shown that AD is accompanied by the deposition of retinal Aβ protein (Grimaldi et al., 2018; Chiquita et al., 2019; Shi et al., 2020), and the retina may have the deposition of Aβ protein before the Aβ accumulation in the brain (Koronyo-Hamaoui et al., 2011; Mirzaei et al., 2019). Aβ protein deposits in the vascular walls, resulting in decreased blood flow, hypoxia and nutrient deficiency. The hypoxic retina promotes angiogenesis by producing vascular endothelial growth factors (VEGF) to ensure essential oxygen and nutrient supplies. However, this process is stopped by the Aβ protein. VEGF is mechanically blocked by the diffuse accumulation of Aβ plaques, and Aβ protein competitively binds to VEGF receptor 2. Therefore, VEGF cannot bind to their corresponding endothelial receptors to restore retinal blood supply to normal levels (Bulut et al., 2018; Yoon et al., 2019). A recent study (Shi et al., 2020) has also suggested that Aβ protein accumulation in retinal blood vessels leads to decreased expression of platelet-derived growth factor receptor-β and pericyte loss, vascular cells that regulate blood flow in capillaries, coupled with decreased expression of LDL receptor-related protein-1(LRP-1), which leads to impaired blood-retinal barrier. The ability to clear Aβ protein is reduced, causing vascular damage. This may be the reason why retinal VD and PD are reduced in patients with AD.

Studies have indicated that the distribution of Aβ protein is not uniform in the retina, but analyzing the changes in the whole retina may ignore the changes in local areas (Lad et al., 2018; Chan et al., 2019). In the present study, the densities of different retinal regions were calculated. After adjusting for confounding factors, the VD and PD in the inner ring (especially in the nasal and inferior regions), and outer ring (especially in the superior and inferior regions) significantly decreased in the AD group compared with HC group. VD and PD in the inner ring (especially in the superior and inferior regions), and outer ring (especially in the superior and temporal regions) significantly decreased in the MCI group compared with HC group. Wu et al. (2020) also conducted a similar study. They also divided the macula into many areas, but their study results showed that the superficial retinal vascular plexus in AD and MCI groups showed no significant difference compared with the normal control group, while the trend of blood flow decline was more obvious in the deep retinal capillary plexus (Wu et al., 2020). But the study did not adjust for confounding factors, and the two studies used different OCTA cameras. This may account for the differences between the two studies. Lahme et al. (2018) also divided the blood vessels of macular retina into superficial layer and deep layer for analysis, and their results were consistent with our results about AD. The blood vessel density in the superficial layer of macular retina in AD patients was lower than that in the control group. Another study also supports our conclusion (Wang et al., 2020). Changes in retinal small vessels may reflect changes in brain small vessels in Alzheimer’s disease. These parameters may be used as alternative non-invasive biomarkers for AD diagnosis. We speculate that the localized changes may be caused by the thinning of ganglion cell layer in AD patients, which changes the retinal blood flow in the corresponding area. The SCP provides nutrients and oxygen to the layers of nerve fiber and ganglion cell in the retina (Jiang et al., 2018). Yoon et al. (2019) investigated the ganglion cell and inner plexiform layer (GC-IPL) thickness in AD patients, and results showed that GC-IPL thickness significantly reduced in AD patients, and the decreased areas were concentrated in the superonasal, inferior and inferonasal regions around the macula. It is roughly consistent with the decreased areas of retinal VD and PD in AD patients in this study. Our study also showed that retinal VD and PD decreased in patients with MCI. This indicates that MCI patients have developed vascular lesions before the onset of clinical symptoms of AD. Therefore, the retinal microvascular network may reflect the early signs of microvascular injury in the MCI and AD patients.

There was no significant difference in the FAZ area between the groups in this study, which was inconsistent with previous findings. Bulut et al. (2018) found that, as compared to healthy controls, the FAZ area in AD patients increased, and a significant negative correlation was noted between FAZ area and MMSE score, suggesting that the lower the MMSE score, the larger the FAZ area is. Similar results were reported by O'Bryhim et al. (2018). In his study, the cognitively healthy subjects were into two groups based on the biomarkers, and results showed significant difference in the FAZ area between two groups, with patients in the biomarker positive group having larger FAZ area (O'Bryhim et al., 2018), but there was no difference in average annual change of FAZ area between the two groups during the 3-year follow-up period (O'Bryhim et al., 2021). Another study showed that the FAZ area remained unchanged (van de Kreeke et al., 2020). But FAZ size varies greatly in healthy people and can be affected by a number of factors (Laatikainen and Larinkari, 1977; Wagner-Schuman et al., 2011; Sampson et al., 2017). Therefore, whether FAZ can be used as a noninvasive retinal marker for AD remains controversial. And more studies with larger sample sizes are needed to confirm the association between FAZ area and AD pathology.

In addition, we compared the correlation between retinal blood flow parameters and cognitive function. MoCA-B and MMSE are usually employed to measure overall cognitive function. HVLT and WMS are used to test the memory function of patients. HVLT-immediate is used to reflect immediate memory, while HVLT-delay is used to reflect delayed memory. BNT and VFT reflect language function. STT tests executive function. ROCFT reflects visuospatial function and memory function. After adjusting for age and sex, overall cognitive function, executive function and visuospatial function were correlated with VD and PD of the retinal SCP. Another study also investigated the correlation between macular retinal blood flow and cognitive function, but no correlation was observed (Yan et al., 2021). The discrepancy between two studies may be ascribed to the differences in diagnostic criteria, statistical methods and OCTA machines. Frontal lobe, temporal lobe and parietal lobe constitute the attention, memory and executive network of the brain (Bero et al., 2011). Regional decrease of cerebral blood flow in AD patients is also mainly manifested in the frontal lobe, temporal lobe, parietal lobe and medial temporal lobe (Kim et al., 2020), suggesting that retinal blood flow may reflect changes in cerebral blood flow. However, this study had a small sample size, and prospective cohort studies with large sample size was still needed to further clarify the correlation between cognitive function and macular blood flow density.

In this study, the CSF was collected from 23 patients and the Aβ protein and p-Tau protein were detected. The correlations of retinal OCTA parameters with CSF Aβ protein and p-Tau protein were further explored. Our results showed no correlation of retinal VD and PD with Aβ protein and p-Tau protein. In a study of Lahme et al. (2018), results also showed no correlation between retinal SCP blood flow density and Aβ protein, p-Tau protein. Whether this implies that AD vascular lesions are primary rather than secondary to Aβ protein deposition and tau protein phosphorylation is still unclear. Therefore, prospective cohort studies with large sample size are needed to further clarify the relationship between retinal blood flow density and pathological proteins.

There were several limitations in the present study. (1) The sample size was small, which may be related to the absence of differences in some parameters. In future studies, we will recruit more patients to expand the sample size. (2) OCTA requires a long shooting time, and patients cannot maintain fixation for a long time, especially patients with severe AD. So our study excluded patients who were unable to cooperate. That’s one of the reasons why we had a low number of patients. (3) The patients were not followed up in this study. This was a cross-sectional study and the dynamic changes of retinal VD and PD were not investigated. In the following study, we will follow up the participants to monitor the dynamic changes in retinal vessel.

In conclusion, retinal SCP microvascular network density reduce in patients with AD and MCI patients as compared to healthy controls, suggesting retinal microvascular dysfunction in MCI and AD patients. Moreover, retinal VD and PD are correlated with some cognitive functional domains. This may be a potential non-invasive biomarker for AD and MCI. Changes in the retinal microvascular network density may offer a valuable insight on the brain in AD.

## Data availability statement

The raw data supporting the conclusions of this article will be made available by the authors, without undue reservation.

## Ethics statement

The studies involving human participants were reviewed and approved by Clinical Research Ethics Committee of Tongji Hospital, Shanghai. The patients/participants provided their written informed consent to participate in this study.

## Author contributions

XM and ZX were responsible for collecting patients’ general information, cognitive information and ophthalmic examination information and writing this article. ZT and HW were responsible for statistical analysis of the data. LZ, YL, and YB were responsible for revising the article. All authors contributed to the article and approved the submitted version.

## Funding

This research was supported by the Shanghai Municipal Health Commission Planning (202240341), Shanghai Hospital Development Center Foundation (Grant No.SHDC12021110), Shanghai Committee of Science and Technology, China(Grant No.22Y11903500), STI2030-Major Projects (2022ZD0211600). Project supported by Clinical Research Project of Tongji Hospital of Tongji University (Grant No. ITJ(ZD)2002.

## Conflict of interest

The authors declare that the research was conducted in the absence of any commercial or financial relationships that could be construed as a potential conflict of interest.

## Publisher’s note

All claims expressed in this article are solely those of the authors and do not necessarily represent those of their affiliated organizations, or those of the publisher, the editors and the reviewers. Any product that may be evaluated in this article, or claim that may be made by its manufacturer, is not guaranteed or endorsed by the publisher.
